# Static nose “Bunny Lines” can be treated using super‐localized phenol‐croton peel: A new approach to an old issue‐case report and discussion

**DOI:** 10.1111/jocd.16497

**Published:** 2024-10-14

**Authors:** G. C. Nogueira, R. I. F. M. Oliveira, M. H. Gold, G. V. Oliveira

**Affiliations:** ^1^ Completa Dermatology Clinic Itaúna Brazil; ^2^ Gold Skin Care Center Nashville USA; ^3^ Santa Casa de Belo Horizonte, Dermatologic Surgery Clinic Belo Horizonte Brazil; ^4^ Faculty of Medical Sciences of Minas Gerais (FCMMG) Belo Horizonte Brazil

To the Editor

Bunny lines, which can include the lower nose areas (Figure 1), are nose horizontal radix wrinkles caused by continuous contracture of the procerus muscle^1^; occipitofrontalis and corrugator supercilii^2^ muscles also account for these contractures. Botulinum toxin has been the main approach for targeting those muscles,^1^ but it may not work to treat deep, static lines. Phenol‐croton peels have been considered one of the gold standard treatments for skin rejuvenation. Croton‐oil is extracted from croton‐tiglium seeds. Phenol leads to the coagulation of the epidermis and it carries croton's pro‐inflammatory molecules to the dermis, leading to skin rejuvenation.^3^ Before Hetter studies, the Baker's formula was the most used deep peel formulation.^4^ Hetter (formula:49.3%phenol and 2.1%croton‐oil)^5^ demonstrated that the active ingredient, croton‐oil,^5^ could vary from 0.2% to 1.6% concentration, allowing for variable concentrations of the peel, depending on the patient's skin's thickness or phototypes. In our clinic, we use the 0,8% Hetter's formula to treat localized areas; with shorter downtime,^6^, ^7^ decreasing the systemic risks of full‐face peels. The purpose of this letter is to describe a reproducible approach successfully employed in our service to treat deep, static nose wrinkles, using a localized phenol‐croton peel.

**FIGURE 1:**
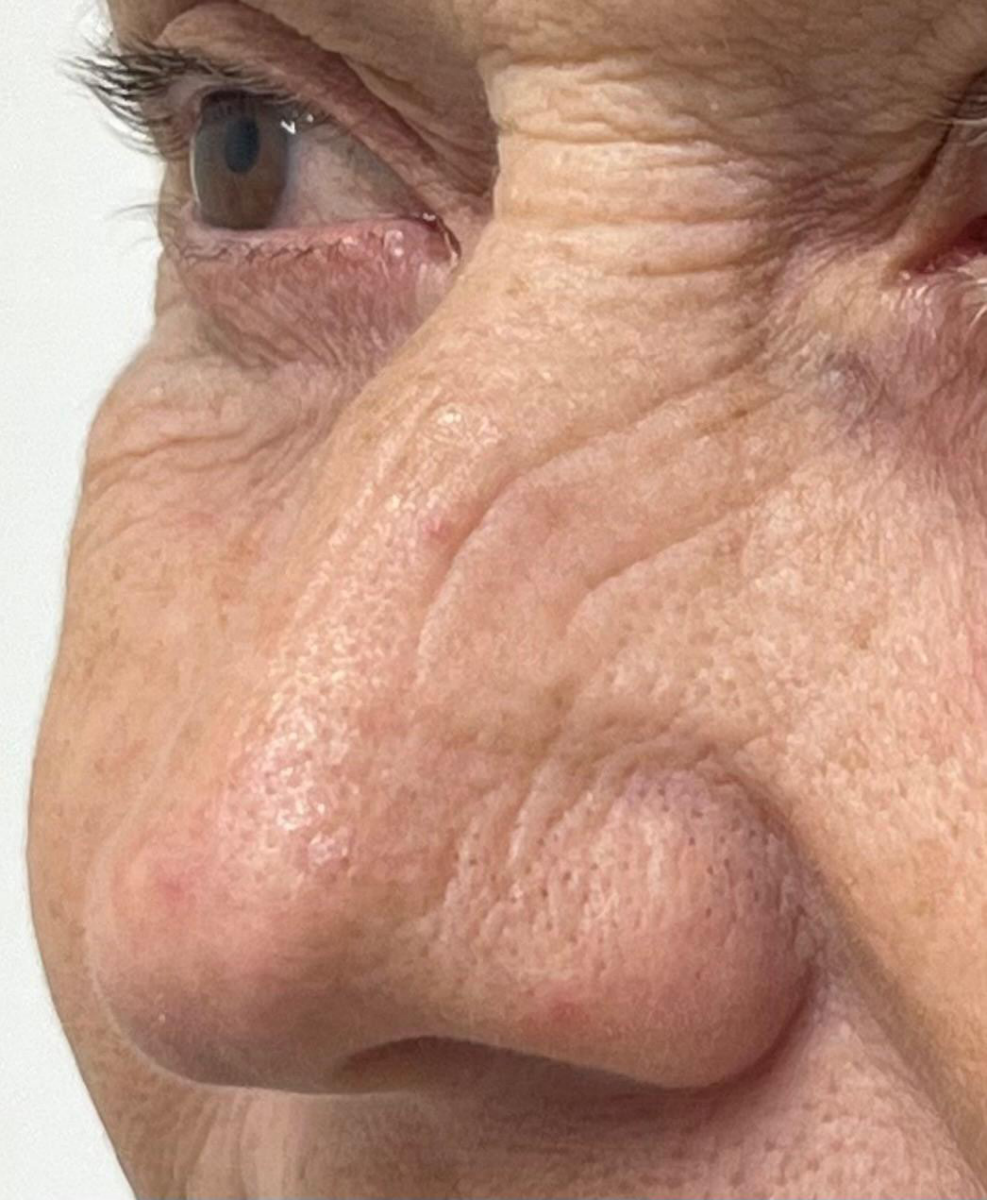
Deep static “bunny lines” continue down to the nose lower areas.

During the procedure, the skin is degreased with a cotton applicator soaked in acetone, and marked with a white pencil. We employ 1% croton‐oil (Hetter's formula), applied with a wooden applicator with cotton at the tip. The applicator is soaked in the solution and passed once over the skin, followed by multidirectional movements with slightly more friction over the wrinkles, without re‐soaking the applicator. The upper limit of the application is the radix, including skin covering the entire bony and cartilaginous part of the nose, including the nasal ala.

No analgesia is required as phenol has an anesthetic effect. We use a fan to relieve any initial burning sensation. After a few minutes, the patient feels a burning sensation in the area, which can last 4–8 h.

Twenty‐four hours later the patient applies silver sulfadiazine and petrolatum jelly to the treated area. After application, the edema in the treatment area reaches its peak around 36 h. Thereafter, it slowly regresses and the skin sloughs off after approximately 7–9 days, resulting in erythematous skin. Erythema persists for 2–4 months. One year and 3 months after the procedure the patient in this case report presented with an outstanding outcome (Figure 2); achieving complete resolution of the deep wrinkles, without demarcation. The patient did not undergo additional treatments, except for home use of retinoic acid and sunscreen.

**FIGURE 2:**
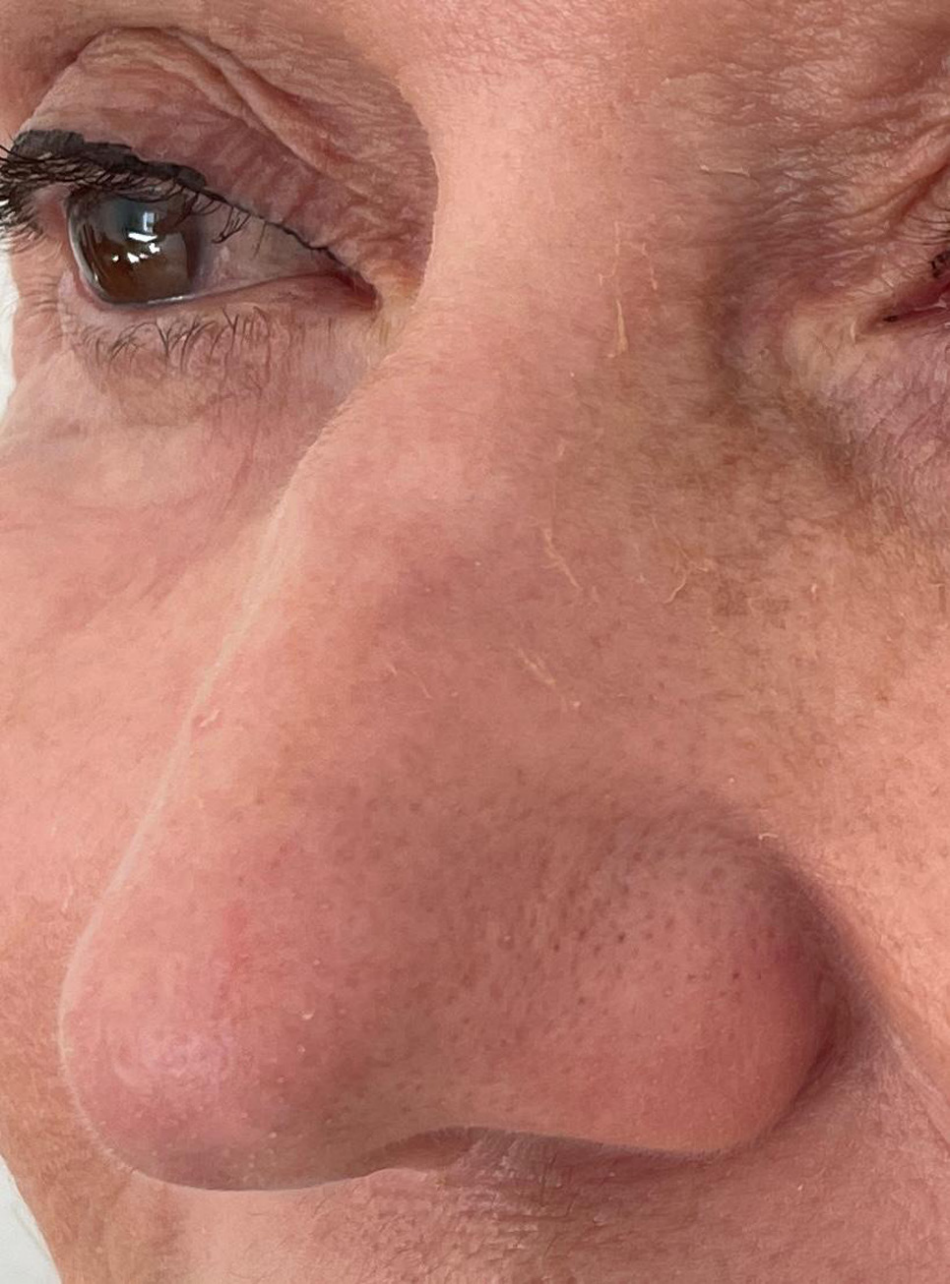
Full improvement of deep nose wrinkles is seen 1.3 months after the localized phenol croton peel on the nose area. Fine desquamation due to the nightly use of retinoic acid can be observed.

Static wrinkles are among the main age‐related facial characteristics of aging, affecting quality of life and psychological wellbeing.^8^ Our group has performed this technique following rhytidoplasty in the operating room, but also as an in‐office procedure, to improve specific areas of concerns, such as the glabella,^7^ the lips and the nose. In over 10 years of practice experience with phenol‐croton peelings, we have observed notable improvement on the nose among patients undergoing full‐face peels, comprising wrinkle reduction, enhanced skin‐quality, decreased pore‐size, and nasal thinning, leading us to start using this technique for localized nose treatments. This technique is a quick, safe procedure with a low risk of demarcation between treated and adjacent areas that can be performed in the office, with long‐lasting results. However, demarcation in the treated areas may occur in patients with higher phototypes or with higher degrees of solar elastosis in adjacent skin. A rapid recovery is expected, due to the large number of appendages in the nose skin.

The “superlocalized‐phenol‐croton peel” may lead to prolonged improvement of horizontal radix lines. Larger studies are needed to evaluate the safety and complication rates for this technique.

## AUTHOR CONTRIBUTIONS

Nogueira GC–technique development; treated patient; manuscript preparation. Oliveira RIFM–technique development; treated patient; literature review. Gold, MH–manuscript preparation; manuscript review. Oliveira GV–literature review; manuscript preparation and review.

## FUNDING INFORMATION

This study has not received funding.

## CONFLICT OF INTEREST STATEMENT

The authors have no conflict of interests to declare.

## CONSENT

The patient gave written consent to the publication of this study. Attachments: patient consent form.

## Supporting information


**Video 1.** The video shows the final aspect of the nose, 15 months after the procedure.

## Data Availability

Data sharing not applicable to this article as no datasets were generated or analysed during the current study.
